# Designing a Simple Electrochemical Genosensor for the Detection of Urinary PCA3, a Prostate Cancer Biomarker

**DOI:** 10.3390/mi15050602

**Published:** 2024-04-29

**Authors:** Meriem Mokni, Amal Tlili, Yassine Khalij, Ghada Attia, Chouki Zerrouki, Wissem Hmida, Ali Othmane, Ali Bouslama, Asma Omezzine, Najla Fourati

**Affiliations:** 1 SATIE Laboratory, UMR CNRS 8029, Cnam, 292 rue Saint Martin, 75003 Paris, France; meriem.mokni@fphm.u-monastir.tn (M.M.); amal.tlili@lecnam.net (A.T.); ghada.attia@lecnam.net (G.A.); chouki.zerrouki@lecnam.net (C.Z.); 2 Biochemistry Department, LR12SP11, Sahloul University Hospital, Route Ceinture Sahloul, Sousse 4054, Tunisia; khalij.yassine@gmail.com (Y.K.); ali.bouslama@rns.tn (A.B.); asmaomezzine2@gmail.com (A.O.); 3 LIMA Laboratory, Faculty of Medicine of Monastir, University of Monastir, Avenue Avicenne, Monastir 5019, Tunisia; ali.othmane54@gmail.com; 4 Sahloul University Hospital, Urology Department, Street Route Ceinture Sahloul, Sousse 4054, Tunisia; hmidawissem@gmail.com; 5 Faculty of Pharmacy of Monastir, University of Monastir, Avenue Ibn Sina, Monastir 5000, Tunisia

**Keywords:** electrochemical genosensor, PCA3 detection, urine samples, prostate cancer diagnosis, affinity constant, specificity testing

## Abstract

This study investigates the feasibility of a simple electrochemical detection of Prostate Cancer Antigen 3 (PCA3) fragments extracted from patients’ urine, using a thiolated single-strand DNA probe immobilized on a gold surface without using a redox probe. To enhance the PCA3 recognition process, we conducted a comparative analysis of the hybridization location using two thiolated DNA probes: Probe 1 targets the first 40 bases, while Probe 2 targets the fragment from bases 47 to 86. Hybridization with PCA3 followed, using square wave voltammetry. The limit of detection of the designed genosenors were of the order of (2.2 ng/mL), and (1.6 ng/mL) for Probes 1 and 2, respectively, and the subsequent sensitivities were of the order of (0.09 ± 0.01) µA^−1^ · µg^−1^ · mL and (0.10 ± 0.01) µA^−1^ · µg^−1^ · mL. Specificity tests were then conducted with the sensor functionalized with Probe 2, as it presents better analytical performances. The electrochemical results indicate that the designed sensor can clearly discriminate a complementary target from a non-complementary one. A further modeling of the calibration curves with the Power Law/Hill model indicates that the dissociation constant increases by one order of magnitude, confirming the ability of the designed sensor to perfectly discriminate complementary targets from non-complementary ones.

## 1. Introduction

Prostate cancer (PCa) stands as one of the most prevalent malignancies globally. As of 2022, it ranked as the second most frequently diagnosed cancer and the fifth leading cause of cancer-related deaths among men, according to the International Agency for Research on Cancer [1].

The early detection of PCa is imperative due to its asymptomatic nature and symptom overlap with benign prostatic conditions [2]. Reliable diagnostic methods are critical for reducing mortality rates and enhancing the efficacy of medical interventions [3,4]. Currently, prostate cancer diagnosis relies on measuring prostate-specific antigen (PSA) levels in serum, followed by digital rectal examination (if PCa is suspected), biopsy, and other radiological assessments. However, the lack of specificity of PSA markers often leads to both false-positive and false-negative results [5,6]. Consequently, various alternative PCa biomarkers, predominantly found in urine samples, have been explored. Urinary biomarkers are valuable tools in clinical research, diagnostics, and patient care across various medical disciplines. They offer several advantages over other types of biomarkers: non-invasive sampling, abundance, stability under a wide range of storage conditions, facilitating ease of handling and transportation compared to more fragile biomarkers found in other bodily fluids, and the reflection of systemic and local changes, as urine contains biomarkers originating from both systemic circulation and local tissues, providing a comprehensive view of physiological and pathological changes in the body [7,8,9].

Several urinary biomarkers were reported as PCa biomarkers, including Engrailed 2 protein, sarcosine, alpha-methyl CoA-racemase (AMACR), and the prostate cancer gene 3 (PCA3), a long noncoding RNA (lncRNA) exclusively produced in prostate cancer tissues [7]. PCA3 expression is reported to be specific to PCa and does not elevate in benign prostatic pathologies, unlike PSA [10,11]. Moreover, numerous studies have demonstrated the correlation of PCA3 levels with tumor volume [12,13], suggesting that PCA3 utilization could significantly reduce the number of unnecessary prostate biopsies. Additionally, PCA3 has been proposed for monitoring the clinical progression of prostate cancer, aiding in therapeutic strategy selection [14,15]. Traditionally, PCA3 detection is performed using RT-qPCR amplification [11,16] or the Progensa test, which is commercially available and approved for clinical use in Europe and the USA [17,18]. However, these tests are time-consuming and expensive and demand highly skilled operators. In recent years, significant progress has been achieved in the advancement of nucleic acid-based electrochemical biosensors, commonly referred to as genosensors. These devices are highly desirable for their ability to provide sequence-specific information in a faster, simpler, and less costly manner than conventional assays such as PCR or RT-PCR. Genosensors find application across a diverse array of fields, including but not limited to the following: neurodegenerative diseases [19,20]; cancerous pathologies encompassing breast cancer [21,22,23], cervical cancer [24,25], gastric cancer [26,27] and colorectal cancer [28]; inherited diseases like coronary artery diseases [29,30], sickle cell anemia [31] and thalassemia [32,33]; and the detection of infectious pathogens (SARS-CoV-2 [34,35], Influenza A virus [36,37], *Haemophilus influenza* [38], and *Mycobacterium tuberculosis* [39,40]).

In the case of PCA3, the literature reported the design of electrochemical genosensors labeled with enzymes [41], redox indicators like methylene blue [42] and ferrocene [43], or nanomaterials for signal amplification [44,45]. Despite its interest, the labeling increases the process’s complexity and cost, necessitating additional time and effort due to intermediate functionalization steps. Moreover, the utilization of electrochemical probes can modify the output signals for several reasons, including variations in probe–analyte interactions, changes in the local environment, which affect electron transfer kinetics, and alterations in the surface properties of the electrode interface [46,47,48].

The demand for effective and portable point-of-care assays has recently surged, presenting new challenges to researchers. Hence, the label-free strategy emerges as an attractive alternative for designing DNA-based electrochemical biosensors [49]. This strategy leverages the intrinsic electrochemical activity of nucleic acids resulting from electrochemically oxidizable or reducible nucleobases [50].

This study proposes a simple label-free method for electrochemically detecting PCA3 extracted from patients’ urine. The procedure involves the generation of both genosensors functionalized with two thiolated DNA probes (Probe 1 and Probe 2) capable of detecting PCA3 through complementarity in different locations: Probe 1 targets the first 40 bases, while Probe 2 targets the fragment from bases 47 to 86. Cyclic voltammetry and square wave voltammetry were investigated to choose the most appropriate technique for PCA3 detection. Once this choice was made, we determined the metrological performances of the genosensors functionalized with the two probes, and we selected the best performing sensor for the specificity tests.

## 2. Materials and Methods

### 2.1. Chemicals and Reagents

Phosphate-buffered saline (PBS), sodium chloride (NaCl), and HEPES binding buffer were procured from Sigma-Aldrich (Lyon, France). Thiolated single-strand DNA (ssDNA) probes and oligonucleotides for non-complementarity (negative control) assays were synthesized by Eurogentec. All reagents utilized for RNA extraction, reverse transcription to cDNA, and PCR (TRIzol^®^, diethyl pyrocarbonate (DEPC)-treated water, forward and reverse primers, Taq polymerase, Moloney Murine Leukemia Virus enzyme, and DNase) were acquired from Invitrogen Thermo-Fisher^®^.

### 2.2. Instrumentation

Urine samples were centrifuged using a Hettich MIKRO 220R centrifuge machine. DNA amplification via polymerase chain reaction (PCR) was conducted in a Proflex^®^ PCR system thermal cycler. The electrophoresis of PCR reaction products was performed using an Apelex^®^ horizontal electrophoresis setup. Images of PCA3 bands revealed on electrophoresis gel were captured using the InGenius^®^ manual gel documentation system. Electrochemical measurements, including square wave voltammetry (SWV) and cyclic voltammetry (CV), were conducted at room temperature and in triplicate using a CHI 650 electrochemical workstation (CH Instrument Inc., IJ Cambria Scientific Ltd., Llwynhendy, UK). A typical three-electrode setup system was employed, with a gold electrode (S = 3.14 mm^2^) serving as the working electrode, a platinum wire as the auxiliary electrode, and (Ag/AgCl) as the reference electrode.

### 2.3. Urine Sample Collection and Processing

This study was approved by the ethics committee of Sahloul University Hospital (Tunisia), and informed consent was obtained from 25 patients scheduled for prostate biopsies. Prior to urine sample collection, digital rectal palpation was performed as described elsewhere [51]. Patients were instructed to void naturally, and the first 20–30 mL of urine were collected and immediately cooled on ice. The samples were processed according to the protocol of Mearini et al. [52]. After centrifugation at 4 °C and 700 rpm for 10 min, urine sediments were collected and subjected to two washes with ice-cold PBS (also by means of centrifugation at 4 °C and 700 rpm for 10 min).

The samples were then preserved at −80 °C in TRIzol^®^ until subsequent use. This reagent, a monophasic solution of phenol and guanidine thiocyanate, serves as a preserving and extraction agent.

### 2.4. Total RNA Extraction from Urine Sediments

Total RNA extraction was performed using TRIzol^®^ according to the supplier’s instructions. Guanidium thiocyanate facilitated cell lysis and the liberation of RNA and DNA. Chloroform was added to aid the partitioning of the aqueous and organic material. RNA was retained in the upper aqueous phase and recovered by means of precipitation with isopropanol, followed by washing with 70% ethanol. RNA pellets were air dried, dissolved in DEPC treated water, and stored in suitable aliquots at −80 °C.

### 2.5. Reverse Transcription of RNA into Complementary DNA

Extracted RNA was treated with DNase to remove the remaining interfering DNA. Reverse transcription was performed using M-MLV enzyme according to the supplier’s protocol, converting RNA into more stable complementary DNA (cDNA) for manipulation and long-term storage.

### 2.6. PCA3 Amplification

PCA3 amplification with the nested PCR method was carried out to enhance the specificity and sensitivity of the reaction. Two pairs of primers were used: pair 1 [53] located in exon 1 and exon 4 of the PCA3 transcript and expected to yield a 543 bp fragment, and pair 2 [16,53] located in exon 3 and exon 4, giving a final 154 bp fragment. Two PCR reactions with pair 1 and then pair 2 were carried out. The final product was resolved on a 2% agarose gel stained with ethidium bromide and visualized using a UV gel documentation system.

**Figure 1:**
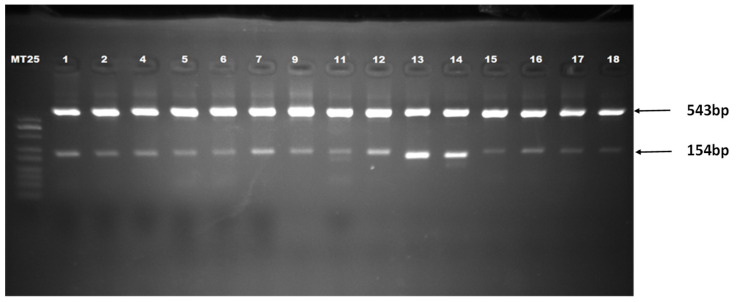
Agarose gel electrophoresis (2%) of amplified PCA3 fragment (Lane MT25: 25 bp DNA ladder; lanes 1 to 15: amplified PCA3 fragments from urine samples).

In addition to the 154 bp fragment expected for this semi-nested PCR, the 543 bp fragment appeared with a higher intensity in all samples tested (Figure 1). This could be attributed to an excess of primer pair 1 in the product of the first amplification, enabling a second amplification of the same fragment. In terms of the obtained results, the 543 bp fragments were selected for further use as real samples for designing the PCA3 genosensor.

Following the manufacturer’s manual, an extraction step of the chosen fragment from the agarose gel was performed using “the Wizard^®^ SV Gel and PCR Clean-Up System”. 

### 2.7. Genosensor Design

The gold electrodes underwent initial cleaning using a polishing process outlined in our previous publication [54]. Two different thiolated single-stranded DNA probes with 40 bases, namely, Probe 1 and Probe 2, were designed to investigate the influence of hybridization location on the detection signal and ensure the specific and selective recognition of PCA3. Using the online tool BLAST (Basic Local Alignment Search Tool), we verified that neither probe cross-hybridizes with any other sequence in the human genome. To ensure complexity while maintaining a unique sequence, we verified that the guanine-cytosine content of both probes is within 35% to 65% [55]. Indeed, the calculated values for Probes 1 and 2 were 52.5% and 41%, respectively. 

**Figure 2:**
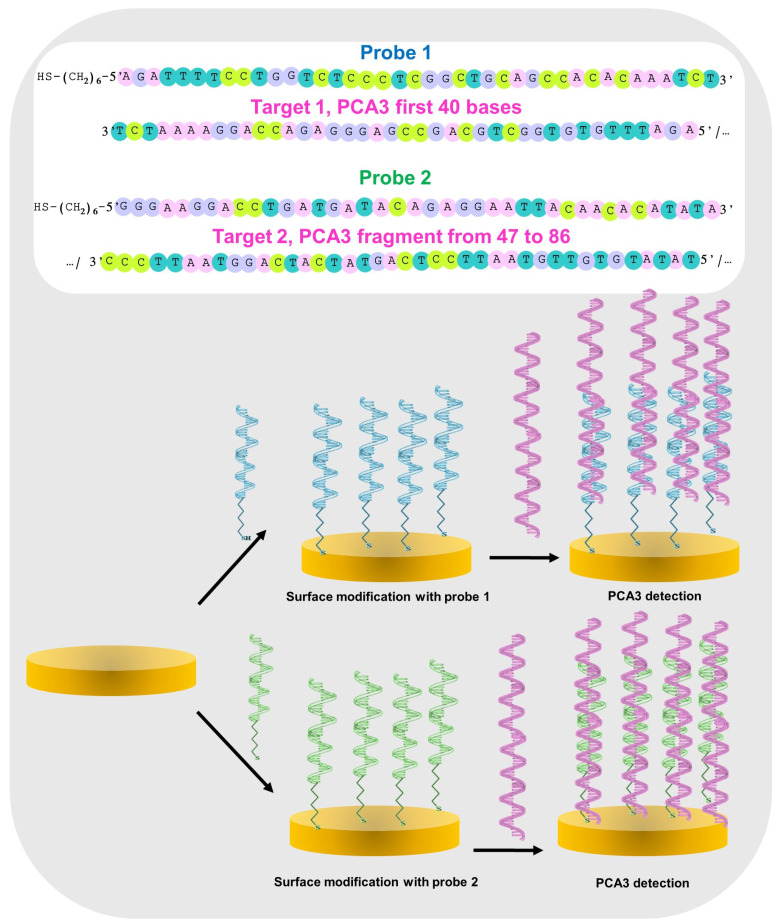
Schematic illustration of the genosensor design, including details of Probes 1 and 2, the functionalization step with the thiolated probes, and a further PCA3 hybridization step.

The sequences of the probes and PCA3 fragment strands (portions 1 and 2) are illustrated in Figure 2 and detailed in Table 1. The Probe 1 sequence could recognize Target 1, the first 40 bases of PCA3, while the Probe 2 sequence recognizes Target 2 (bases 47 to 86 of the PCA3 fragment).

It should be noted that thiol-modified DNA strands demonstrate an increased regularity in orientation upon immobilization on a gold surface. This enhanced regularity is due to multiple factors, including the strong affinity between thiol groups and gold and the repulsive van der Waals forces between the DNA chains [56,57,58]. These factors collectively contribute to the orderly arrangement of DNA molecules on the gold surface, resulting in enhanced orientation regularity, as depicted in Figure 2.

The thiolated probes were initially diluted to achieve a final concentration of 20 μg/mL (1.6 μM) in a 0.3 M NaCl solution. Covalent immobilization was then carried out by depositing 10 µL of the probe solution onto the gold surface and incubating it at room temperature for 1 h. This duration was optimized in our previous work dedicated to the design of a DNA biosensor [29]. The operation was carried out identically for the two probes separately. The electrodes were then rinsed with PBS. Subsequently, the surface underwent reincubation for 1 h with various concentrations of PCA3 pre-treated with heating at 95 °C and then immediately cooled in an ice bath to obtain single-stranded fragments. 

Cyclic voltammetry (CV) and square wave voltammetry (SWV) techniques were employed to monitor the surface modification and PCA3 detection process. For CV, a sweep cycle ranging from −0.6 to 0.6 V was applied at a scan rate of 100 mV/s. For SWV measurements, the parameters were as follows: a potential range from −0.6 to 0.6 V, a voltage increment of 4 mV, an amplitude maintained at 25 mV, a waveform frequency set to 25 Hz, and a sensitivity set to 10^−3^.

## 3. Results

### 3.1. Optimization of the Electrochemical Parameters for the Genosensor’s Design

Each of the electrochemical parameters was separately optimized based on surface modification with Probe 1, which is used as a model in this section. Cyclic voltammetry (CV) was chosen to monitor the electrochemical sensor response before and after the addition of various concentrations of PCA3. The measurements were conducted in a 0.3 M NaCl solution without adding any label by applying a sweep cycle from −0.6 V to 0.6 V at a scan rate of 100 mV/s. Using the ionic strength of 0.3 M in our study was based on the study conducted by Revenga-Parra et al. [59], who indicated that higher ionic strengths can enhance the repulsion between DNA probes during both pre- and post-hybridization phases, thereby enhancing sensor stability. Additionally, this choice is supported by our previous research [29], in which we demonstrated that this ionic strength yields favorable sensor performance results.

**Figure 3:**
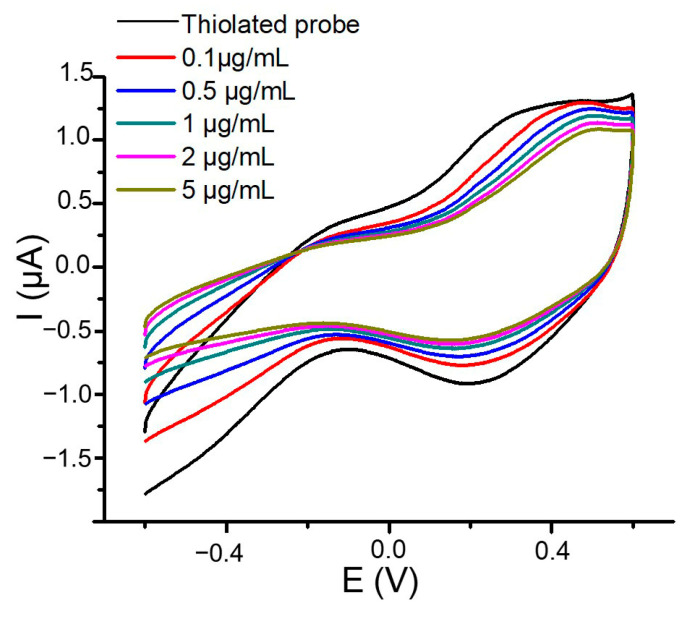
Cyclic voltammograms corresponding to the grafting of the thiolated Probe 1 on a gold surface and further hybridization with different concentrations of PCA3 strands. All measurements were performed in a 0.3 M NaCl solution.

The voltammogram displayed in Figure 3 shows an electrochemical nucleic acid oxidation potential at 0.49 V and a reduction potential at 0.19 V. These findings align with previous studies by Paleček and colleagues [60,61], who demonstrated the possible oxidation of adenine and guanine bases and the reduction of adenine, cytosine, and guanine. Consequently, an electrochemical analysis of DNA can be conducted based on its intrinsic electroactivity without adding any labels or redox indicators [50]. Afterward, the surface underwent a 1 h incubation period with varying concentrations of PCA3 pre-treated with heating at 95 °C, followed by immediate cooling in an ice bath. This procedure was intended to generate single-stranded fragments.

The data in Figure 3 illustrate a decrease in the oxidation and reduction current peak absolute values when PCA3 concentrations increase from 0.1 μg/mL to 5 μg/mL. This variation is likely attributed to the formation of double-stranded DNA on the surface, resulting from target hybridization. Previous studies have noted that the voltammetric responses of double-stranded DNA are lower than those of single-stranded DNA because of the inaccessibility of the electroactive sites of guanine, cytosine, and adenine located within the duplex [50,62,63,64]. Additionally, in double-stranded DNA, some phosphate groups are concealed inside the helix, making them less accessible to the solvent and ions, thus slightly reducing their contribution to the effective negative charge carried by the medium.

**Figure 4:**
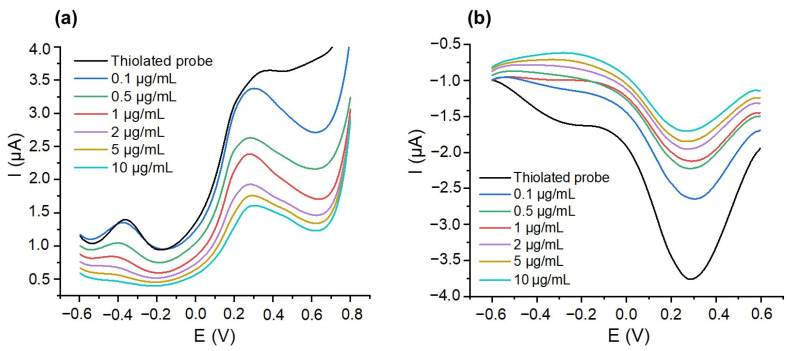
Square wave voltammograms corresponding to the grafting of the thiolated Probe 1 on a gold surface and further hybridization with different concentrations of PCA3 strands (**a**) in oxidation mode and (**b**) in reduction mode. All measurements were performed in a 0.3 M NaCl solution.

However, the low resolution of the CV spectra in both oxidation and reduction warrants further investigation. Square wave voltammetry (SWV), known for reducing capacitive background, was thus employed to address this. SWV characterizations were monitored in oxidation and reduction modes by sweeping the potential from −0.6 V to 0.6 V and from 0.6 V to −0.6 V, respectively. The results presented in Figure 4 show that SWV voltammograms are better resolved than CV ones, confirming the ability of this electrochemical technique to enhance output signals.

**Figure 5:**
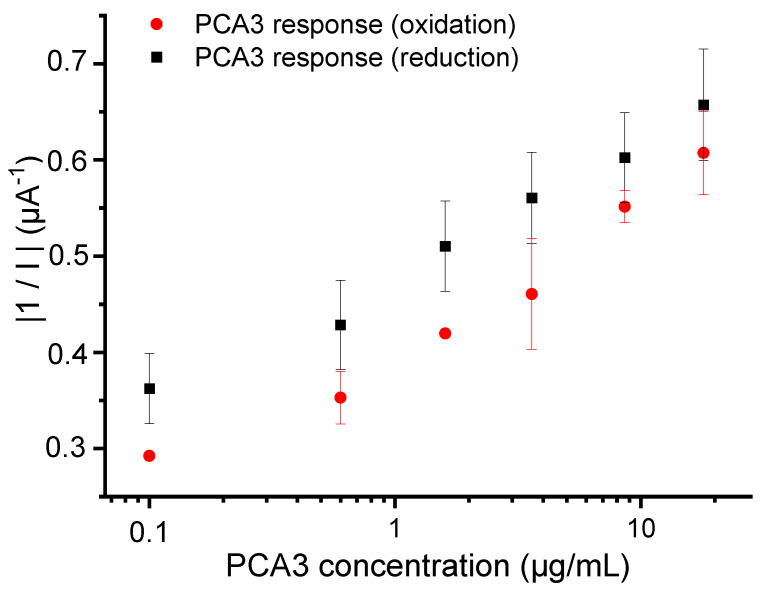
Semi-logarithmic variations of the absolute value of 1/I versus cumulative PCA3 concentrations obtained from SWV measurements in oxidation and reduction modes. All measurements were performed in a 0.3 M NaCl solution.

To enhance the visualization of this variation, our next step involved plotting the absolute value of 1/I against PCA3 concentrations (Figure 5).

The sensitivities, calculated from the slope of the calibration curve, were comparable, of the order of (0.08 ± 0.01) and (0.09 ± 0.01) µA^−1^ · µg^−1^ · mL for oxidation and reduction, respectively. Nevertheless, the measurements conducted in SWV reduction mode yielded the most intense output signals, with good reproducibility. To elucidate this finding, we calculated the percentage (P%) of oxidative and reductive nucleic acid in the thiolated probe using Equation (1). According to the literature [60,61], adenine and guanine bases are susceptible to oxidation, while adenine, cytosine, and guanine can undergo reduction processes. Based on this, we calculated the number of nucleotides that are oxidized or reduced per strand, and then we divided this number by the total number of nucleotides. This normalization process allows us to determine the proportion of oxidized or reduced bases relative to the total nucleotide composition:

(1) $$P\%=\frac{Number\;of\;oxidized\;(or\;reductive)\;nucleic\;acids}{Total\;number\;of\;nucleic\;acids\;in\;the\;probe}\times 100$$

*P*% was found equal to 37.5% and 72.5% for oxidative and reductive nucleic acid, respectively. This result highlights the influence of the probe sequence and structure in enhancing electrochemical signals. SWV measurements in reduction mode were therefore selected for subsequent investigations.

### 3.2. Influence of Probe’s Type on the Hybridization Process

**Figure 6:**
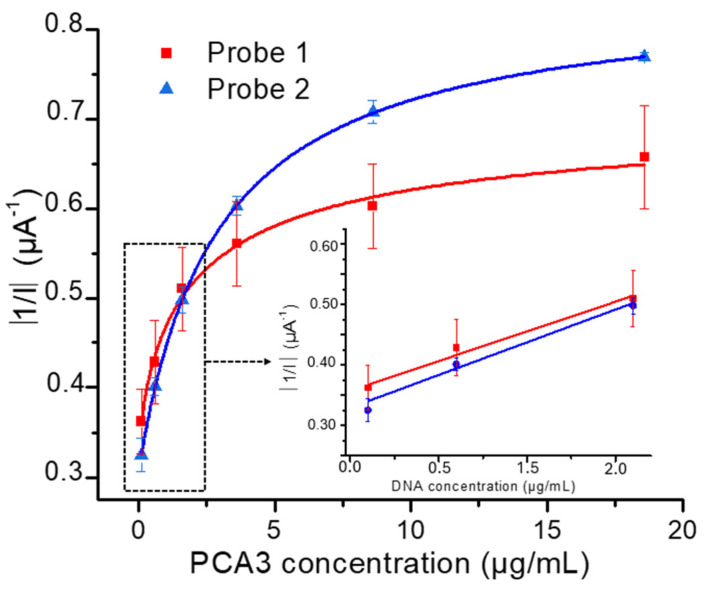
Genosensor calibration curves. The inset represents the linear mode for sensitivity calculations.

Two sensors, functionalized with two different probes, Probe 1 (complementary to portion 1) and Probe 2 (complementary to portion 2), were designed to investigate the impact of probe structure on the hybridization process and to select the most suitable one in terms of signal intensity, detection limit, and sensitivity. PCA3 concentrations varied from 0.1 μg/mL to 10 μg/mL. The corresponding calibration curves, representing the absolute value of 1/I variations versus the cumulative concentrations of PCA3, are plotted in Figure 6. 

The precision for the output signal for Probes 1 and 2 was estimated to be of the order of 90% and 94%, respectively. The limit of detection (LOD) was calculated (Equation (2)) based on the IUPAC (International Union of Pure and Applied Chemistry) method, which quantifies the minimum detectable concentration of a substance based on the variability of the blank signal and the slope of the regression line, and is typically expressed as:

(2) $$LOD={3\times SD}_{blank}/Slope$$

where *SD_blank_* is the standard deviation of the blank signal, and *Slope* is the slope of the regression equation within the linear range from 0.1 µg/mL to 1 µg/mL.

The LOD values were of the order of 2.2 ng/mL (13.1 pM) and 1.6 ng/mL (9.1 pM) for the sensors functionalized with Probe 1 and Probe 2, respectively. These values are lower than previous work reported in the literature (Table 2). These results are highly encouraging, demonstrating that the genosensor can effectively detect the biomarker PCA3 at concentrations as low as 0.12 µg/mL. This sensitivity is particularly significant for the early diagnosis of prostate cancer [65].

The sensitivity values, calculated from the slopes at the origin of the calibration curves, were estimated at (0.09 ± 0.01) and (0.10 ± 0.01) µA^−1^ · µg^−1^ · mL for the sensors functionalized with Probe 1 and Probe 2, respectively. These results indicate that the genosensor functionalized with Probe 2 is slightly more sensitive than that modified with Probe 1.

Taking into account all these findings, Probe 2 emerges as the most promising candidate due to its highest output signal, superior sensitivity, and lowest limit of detection (LOD).

To understand the reasons for this variation, a complementary study was initiated to explore the potential generation of secondary structures in both probes. This investigation utilized the Mfold online tool developed by Zuker [68]. The simulation yielded ΔG values of −4.16 kJ/mol and −1.83 kJ/mol for Probe 1 and Probe 2, respectively. Since negative ΔG values signify a thermodynamically favorable process, loop formation is thus energetically favored for both probes. 

**Figure 7:**
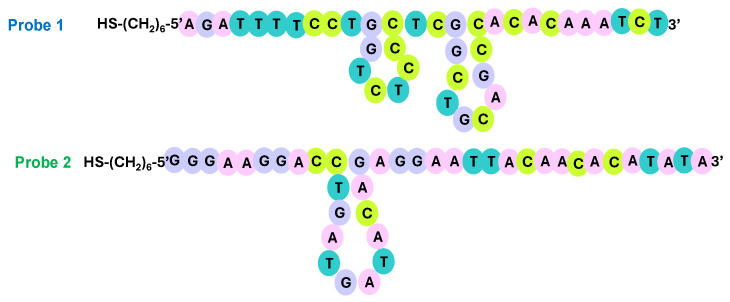
The folded probes’ structures generated using the Mfold online tool simulating experimental conditions (DNA at 25 °C, [Na^+^] = 0.3 M).

Figure 7 illustrates the probability of loop formation for each probe. Probe 1 exhibits the potential to fold into two loops, while Probe 2 could only generate one loop.

As suggested by Ermini et al. [69], the formation of secondary structures like hairpins or loops might reduce hybridization and inhibit the recognition and the synergy between the genosensor and PCA3. Consequently, Probe 2 emerged as the more suitable option for PCA3 detection and was chosen for subsequent investigations.

### 3.3. Specificity Tests

One of the primary critical parameters in the design of a genosensor is its specificity, which assesses the sensor’s capacity to distinguish between complementary and non-complementary strands. To the very best of our knowledge, specificity tests have not been reported in any studies dedicated to the detection of PCA3. 

The specificity test was carried out against four non-complementary (NC) oligonucleotides, NC1, NC2, NC3, and NC4, whose sequences are detailed in Table 3. These oligonucleotides were meticulously chosen during our investigation. We analyzed non-complementary (NC) strands from several genes: NC1 and NC2 from the PCA3 gene, NC3 from the PSA gene, and NC4 from the beta-2 microglobulin gene, all of which are present in men’s urine. 

**Figure 8:**
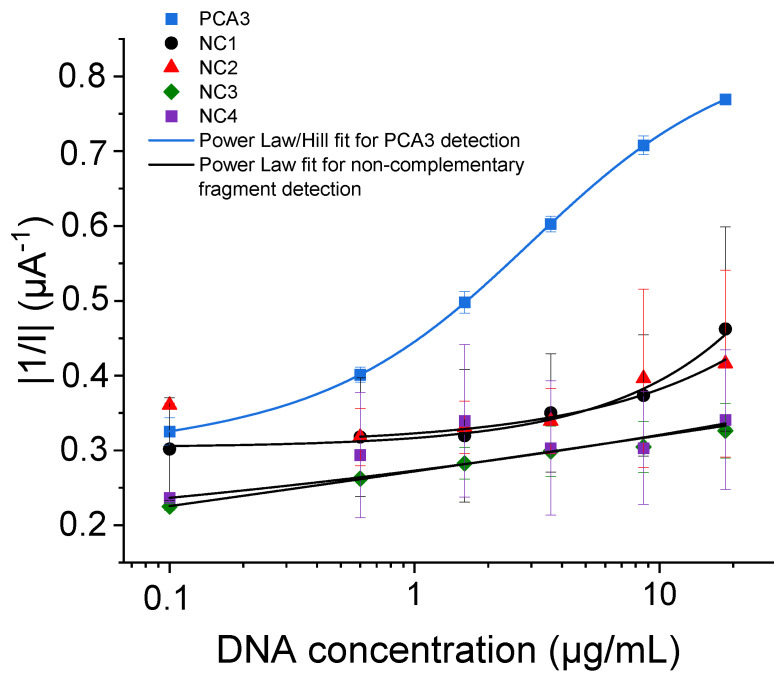
Semi-logarithmic variations of the absolute value of 1/I variations versus cumulative concentrations of PCA3 and fragments NC1, NC2, NC3, and NC4.

The electrochemical answer of the genosensor against the non-complementary fragments is depicted in Figure 8. This figure illustrates that the output signal from PCA3 hybridization is approximately 57% higher than that obtained with non-complementary fragments. It is also important to note that the signal from the non-complementary fragments exhibits a high error margin, possibly due to the random and non-specific interactions between Probe 2 and the non-complementary fragments. 

To understand the affinity between PCA3 and Probe 2, the dissociation constant (*K_d_*) was estimated using a combined power law and Hill model, as outlined in Equation (3). The power law component models non-specific adsorption, while the Hill equation component accounts for specific recognition processes [54,70]. This approach provides a comprehensive analysis of both non-specific and specific interactions between PCA3 and the probe:

(3) $$S\left(C\right)=(A+B\times C^{x})+\frac{E\times C^{n}}{K_{d}^{n}+C^{n}}$$

where *S* is the sensor output signal expressed in the absolute value of 1/I, *C* is the concentration, *A*, *B*, and *E* are empirical constants, *x* is a weighting exponent, *n* is the Hill coefficient, and *K_d_* is the dissociation constant. 

The analysis of these experimental data reveals significant insights into the behavior of the genosensor in detecting PCA3. Firstly, the combined model, integrating power law and Hill components, has proven to be highly effective. This model accurately fits the experimental data from the PCA3 calibration curve, yielding a dissociation constant of (2.97 ± 0.05) μg/mL. This precise fit underscores the model’s ability to capture the specific interactions between PCA3 and the probe, validating its use in quantifying PCA3 concentrations. Secondly, when assessing the response of the genosensor to non-complementary fragments, the “power-law” model emerged as the most suitable. It effectively describes the genosensor’s behavior, closely matching the experimental observations and thereby confirming its appropriateness for modeling non-specific adsorption events. 

After that, we calculated the corresponding sensitivities to further elucidate the specificity of the DNA sensors. The results, presented in Table 4, confirm the reliability of the designed sensor in differentiating between specific and non-specific binding events.

## 4. Outlook

The DNA-based biosensor designed in the framework of this study can be used for point-of-care testing (POCT) and could fundamentally change the landscape of early prostate cancer detection. This non-invasive diagnostic tool would also be particularly valuable for monitoring disease progression and recurrence, allowing for timely adjustments to treatment plans. Moreover, by offering a more precise risk assessment than traditional PSA tests, the designed PCA3 biosensor could significantly reduce the number of unnecessary biopsies, which are invasive and often lead to complications. Integrating microfluidic technology with electrochemical detection will streamline sample processing and reduce manual handling, which is crucial for making the test user-friendly and rapid at the point of care.

## 5. Conclusions

This study presents the development of an innovative and sensitive electrochemical genosensor, specifically designed for the detection of PCA3 RNA fragments from urine samples. The sensor was functionalized with thiolated single-strand DNA probes covalently attached to gold electrode surfaces to identify specific segments of the PCA3 sequence. In our methodology, we utilized two distinct DNA probes: one targeting the initial 40 nucleotides and the other spanning nucleotides 47 to 87. The electrochemical detection was performed using square wave voltammetry (SWV) in reduction mode, which proved to be the most effective technique for our application. This study demonstrates that the genosensor functionalized with the second probe significantly outperformed the first, exhibiting a lower limit of detection (1.6 ng/mL versus 2.2 ng/mL) and a higher sensitivity. Specificity tests with Probe 2 confirmed the ability of the genosensor to distinguish between complementary and non-complementary DNA oligonucleotides.

Despite these promising results, the designed PCA3 genosensor faces a limitation that could hinder its deployment as a point-of-care testing (POCT) device, namely, the need for the pre-amplification of RNA targets, which adds complexity and may affect the feasibility of implementing these sensors in a typical clinical setting.

To address this challenge and enhance the utility of PCA3 genosensors in POCT applications, future improvements could include the integration of advanced signal amplification strategies, such as the use of nanomaterials to enhance the sensitivity and reduce the limit of detection. Moreover, developing multiplexed sensors capable of simultaneously detecting multiple biomarkers, such as PCA3 and PSA mRNA, would provide a more comprehensive diagnostic tool, thereby increasing the accuracy and reliability of prostate cancer diagnosis.

## Figures and Tables

**Table 1:** Sequences of Probes 1 and 2 and their complementary strands.

	Sequence
Probe 1	H-S-(CH_2_)_6_-5′-AGATCTTCCTGGTCTCCCTCG GCTGCAGCCACACAAATCT-3′
Probe 2	H-S-(CH_2_)_6_-5′-GGGAAG GAC CTG ATG ATA CAG AGG AAT TAC AACACATATA-3′
Target 1	5′-AGATTTGTGTGGCTGCAGCCGAGGGAGACCAGGAAGATCT-3′
Target 2	5′-TATATGTGTTGTAATTCCTCTGTATCATCAGGTCCTTCCC-3′

**Table 2:** The performance characteristics of PCA3 genosensors.

Surface Modification	Redox Probe Use	Concentration Range	LOD pM	Ref.
RNA aptamer	Ferrocene	0.1 × 10^−3^ to 1 µg/mL	400	[43]
Thiolated DNA aptamer	Methylene blue	0 to 150 ng/mL	9200	[66]
CHT/MWCNT/SsDNA	K_3/4_[Fe(CN)_6_]	10^−16^ to 10^−6^ M	1280	[45]
RNA aptamer	Ferrocene	0 to 10 nM	10	[67]
AuNPs/chondroitin sulfate/SsDNA	K_3/4_[Fe(CN)_6_]	10^−6^ to 10 μM	83	[44]
Thiolated DNA (probe 1)	Without redox probe	0.1 to 10 µg/mL	13	The present work
Thiolated DNA (probe 2)	Without redox probe	0.1 to 10 µg/mL	9	

**Table 3:** Sequences of the non-complementary fragments used for specificity tests.

	Sequence	Description
NC1	5′-AGATTTGTGTGGCTGCAGCCGAGG GAGACCAGGAAGATCT-3′	The first 40 nucleotides of the amplified PCA3 fragment
NC2	5′-GATGACCCAAGATGGCGGC-3′	A PCA3 portion from bases 486 to 507
NC3	5′-CCTCCTGAAGAATCGATTCCT-3′	A portion from PSA gene
NC4	5′-ATGGATGAAACCCAGACACA -3′	A portion from beta-2 microglobulin gene

**Table 4:** Comparative table of the sensor’s sensitivities for PCA3 and non-complementary oligonucleotides.

	Sensitivity (µA^−1^·µg^−1^·mL)
PCA3	0.10 ± 0.01
NC1	0.011 ± 0.007
NC2	0.006 ± 0.002
NC3	0.014± 0.002
NC4	0.07 ± 0.01

## Data Availability

The original contributions presented in the study are included in the article, further inquiries can be directed to the corresponding author.
